# Supplementary material for: A 4-Gene Signature of CDKN1, FDXR, SESN1 and PCNA Radiation Biomarkers for Prediction of Patient Radiosensitivity
Source: Int J Mol Sci. 2021 Sep 30;22(19):10607. doi: 10.3390/ijms221910607 (PMC8508881; doi:10.3390/ijms221910607)
Supplement: Supplementary file 1 [file ijms-22-10607-s001.zip › ijms-1381108-supplementary.pdf]

Supplementary Table 3: Clinical details of prostate cancer donor cohort at baseline (pre-hormone and pre-radiotherapy (RT) treatment) derived from CTRIAL-IE (ICORG) 08-17

Prostate Patient code	Age at consent	TMN*	Gleason Score	Hormone regime	RT regime	KPS%**	Referring PSA***	RT Toxicities (Baseline)^	RT Toxicities (Post RT)^^
**PC1**	65	T3aN0M0	3+4=7	Prescribed x 3yrs	81Gy/45#	100%	6.6	Gr 1 urinary frequency	Gr 1
**PC2**	71	T3aN0M0	4+5=9	Prescribed x 3yrs	81Gy/45#	100%	7.43	Gr1 urinary urgency/frequency (urg/freq), urinary retention, urinary incontinence. Nocturia = 2 times per night.	Gr 1
**PC3**	65	T3bN0M0	4+4=8	Prescribed x 3yrs	81Gy/45#	100%	6	Gr1 fatigue,urinary urg/freq Nocturia 4= times per night.	Gr 1
**PC4**	85	T3aN0M0	4+5=9	Prescribed x 3yrs	81Gy/45#	90%	11.9	**NR**	
**PC5**	60	T2cN0M0	4+4=8	Prescribed x 3yrs	81Gy/45#	90%	11.38	Gr1 Fatigue, dysuria, urinary retention, cystitis; Gr3 urinary urg/freq. Nocturia 8 = times per night.	Gr 1
**PC6**	68	T2cN0M0	3+5=8	Prescribed x 3yrs	81Gy/45#	90%	6.2	Gr1 urinary retention. Nocturia = 2 times per night	Gr 2 urinary incontinence
**PC7**	58	T3aN0M0	4+5=9	Prescribed x 3yrs	81Gy/45#	100%	9.08	Gr1 Fatigue, urinary urg/freq. Nocturia = 1 time per night	Gr 2 urinary urg/freq
**PC8**	63	T3aN0M0	4+5=9	Prescribed x 3yrs	81Gy/45#	100%	8.2	Gr1 urinary urg/frequency. Gr2 erectile dysfunction. Nocturia = 1 time per night	Gr 1
**PC9**	74	T3bN0M0	3+4=7	Prescribed x3 yrs	81 Gy/45#	100%	5.3	Gr1 constipation, urinary retention. Nocturia = 2 times per night.	Gr 2 urinary urg/freq, nocturia

**PC10**	57	T3BN0M0	4+5=9	Prescribed x 3yrs	81Gy/45#	100%	21.00	Gr1 urinary urg/freq, urinary retention. Nocturia = 2 times per night.	Gr 2 urinary urg/freq
**PC11**	67	T3aN0M0	3+4=7	Prescribed x 3yrs	81Gy/45#	100%	9.24	Gr1 libido. Gr2 erectile dysfunction.	G2 urinary urg/freq, nocturia
**PC12**	70	T3aN0M0	3+4=7	Prescribed x6mth	81Gy/45#	100%	**NR**	Gr1 urinary urg/freq. Nocturia= 1 times per night	Gr 1
**PC13**	75	T2bN0M0	4+4=8	Prescribed x6mth	81Gy/45#	100%	19.57	Gr1 urinary urg/ freq, libido. Gr3 erectile dysfunction. Nocturia = 2 times per night.	Gr 1
**PC18**	69	T2aN0M0	4+4=8	Prescribed x 3yrs	81Gy/45#	100%	49.06	Gr1 libido, Gr3 erectile dysfunction. Nocturia= 1 times per night	Gr 1

Notes: NR refers to Not Recorded, due to prostate cancer samples that were derived from different local hospitals.

***TNM**: Tumor Node Metastasis staging system used to classify prostate tumours in patients.

****KPS%:** Karnofsky performance score (KPS) used to assess prostate patients ability to perform ordinary tasks. Values >80% indicative of ability to perform normal tasks and work with no special care needed.

*****Referring PSA:** Prostate Specific Antigen (PSA) is measured in the blood of prostate cancer patients in nanogram per mililitre (ng/ml) at referral stage. The normal reference range for cohort is 3.9ng/ml (50-59yrs), 4.5ng/ml (60-69yrs), 5.5ng/ml (>70yrs) and therefore PSA values exceeding the reference range are indicative of prostate cancer.

^RT Toxicities: Genitourinary toxicities recorded at baseline at Grade 1 (Gr1) and/or Grade 2 (Gr2). Minimal toxicity at Gr 0-1, high toxicity at Gr 2+

^^ RT Toxicities (post RT): Genitourinary toxicities recorded 2 months post radiotherapy treatment.

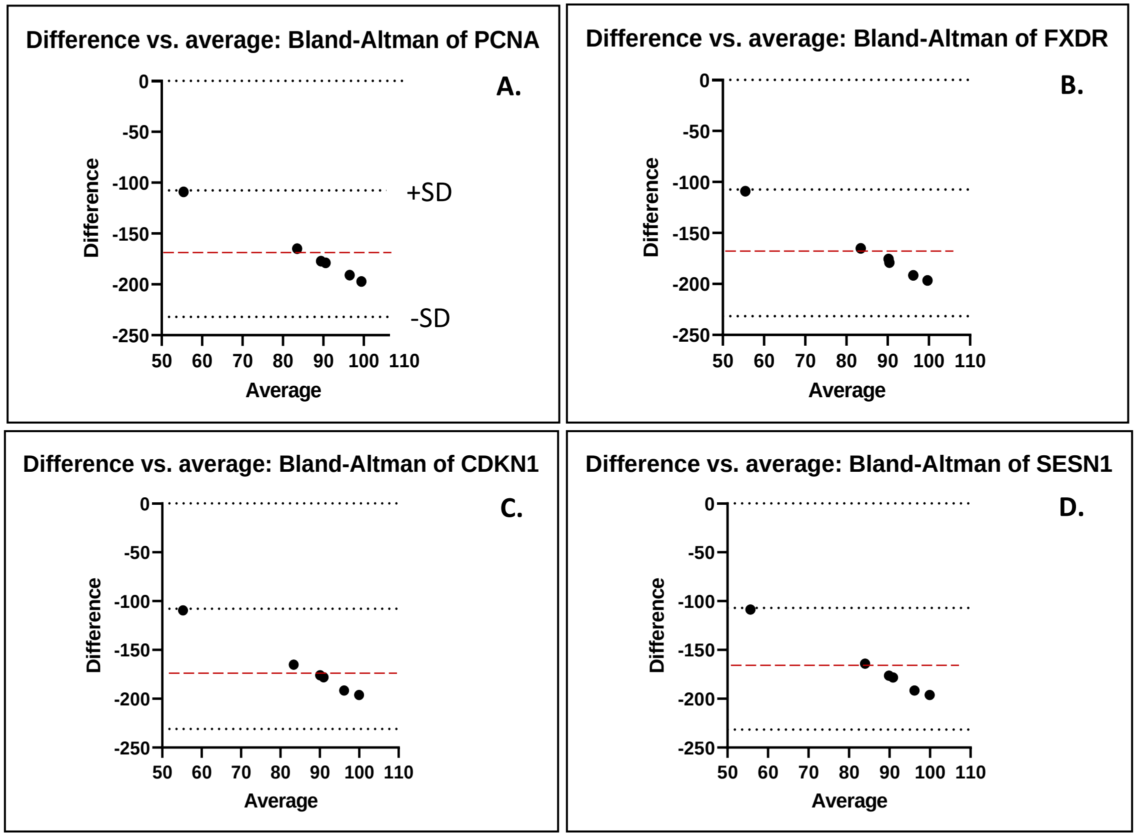

Supplementary Figure 6. Bland-Altman plots of G2 Radiosensitivity scores compared with gene expression at 0.5Gy for each of the 4 genes; PCNA (A), FXDR (B), P21 (C) and SESN1 (D). The 95% levels of agreement are indicated by the +SD and –SD dotted lines indicated on PCNA (A) and for each graph B-D where 95% of the data points fall. The mean difference (bias) is indicated as the red line in between the 95% level of agreement for each graph and was similar for all 4 genes. Specifically, -169.7 (A), -169.5 (B), -169.4 (C) and -169.2 (D).
